# Emerging Therapies in Penile Cancer

**DOI:** 10.3389/fonc.2022.910335

**Published:** 2022-06-21

**Authors:** Antonio Machado Alencar, Guru Sonpavde

**Affiliations:** ^1^ Grupo de Estudos em Patologia Molecular, Hospital Universitário da Universidade Federal do Maranhão, São Luís, Brazil; ^2^ Department of Clinical Oncology, Hospital São Domingos/Dasa, São Luís, Brazil; ^3^ Department of Genitourinary Oncology, Dana-Farber Cancer Institute, Boston, MA, United States

**Keywords:** penile cancer, human papilloma virus, chemotherapy, immune therapy, targeted therapy

## Abstract

Advances in the treatment of rare tumors like penile cancer were always hampered by the lack of deep comprehension of the molecular biology and genomic and epigenomic alterations involved in carcinogenesis and tumor progression, as well as by the difficulty in recruitment of patients for prospective clinical trials. Despite the high rates of cure in early localized penile cancers with surgery or other local procedures, locally advanced and metastatic tumors require systemic treatment, with chemotherapy being the current standard, but with high toxicity and no proven real impact on survival. Recent important findings of frequent genomic alterations and mutation signatures in penile cancer have motivated several trials in new modalities of systemic treatments, especially immunotherapy. This review aims to present the most recent advances and the prospect of new modalities of systemic therapies with ongoing studies in penile cancer.

## Introduction

Penile cancer is a rare disease with a total number of cases estimated at 36,068 globally in 2020 (0.92 cases/100,000 people) (1). However, these tumors have a higher incidence in developing countries, reaching up to 6.1 cases/100,000 people (2). The most frequent histology, responsible for almost the totality of cases, is squamous cell carcinoma (SCC). Overall survival (OS) in early disease without nodal involvement is 96% in 10 years with curative surgery (3), while 5-year median OS of patients with regional node disease and distant metastatic disease are, respectively, 50% and 12% (4).

Cytotoxic chemotherapy plays a key role in the systemic treatment and consists mainly of platinum and taxane combination regimens based on the results of small phase II trials, with typical chemotherapeutic toxicities and modest survival outcomes, both in advanced disease treatment (5, 6) and in the neoadjuvant setting (7, 8). There are no prospective randomized trials that address this issue. In the adjuvant and neoadjuvant scenario, the real role and better sequence of multimodal treatment with radiotherapy, surgery, and chemotherapy in patients with operable nodal involvement are still under investigation in the ongoing phase III International Penile Advanced Cancer trial (InPACT) study (NCT02305654).

There is an urgent need for more efficient and less toxic new modalities of systemic treatment for advanced penile SCC based on the current knowledge of its molecular pathogenesis, including targeted therapy, immunotherapy, and new classes of drugs and combinations regimens that can meet this demand. This review displays the current therapies available and the perspective of novel therapies under investigation.

## Current Standard of Systemic Treatment: Cytotoxic Chemotherapy

Cytotoxic chemotherapy, based on different combinations that include platinum, 5-FU, taxanes, and ifosfamide remains the mainstay of systemic treatment. For patients with locally advanced disease (T3N+, T4, or N2/N3) overall response rate (ORR) with neoadjuvant chemotherapy varies from 50% to 60% (7, 8). The most recommended combination is paclitaxel, ifosfamide, and cisplatin (TIP). In a phase II trial, 30 patients with N2 or N3 disease were treated with neoadjuvant TIP and pathologic complete response occurred in 10%. Surgery was performed in 73.3% of patients and the median OS was 17.1 months (7). A different drug combination containing paclitaxel, 5-FU, and cisplatin (TPF) was evaluated in a phase II trial that included 26 patients with a successful surgery rate of 53% and median OS of 10.1 months (8). However, there are no phase III trial results to date that supports the use of neoadjuvant chemotherapy, and the rate of grade 3 toxicity of neoadjuvant chemotherapy containing taxanes is 49% (9). The InPACT (NCT02305654) is the first phase III trial of neoadjuvant chemotherapy in penile cancer and its results are expected in July 2022. This trial evaluates the role of neoadjuvant chemotherapy with or without radiation before surgery and the role of prophylactic pelvic lymph node dissection in those receiving adjuvant chemoradiation for high-risk inguinal node-positive disease. Regarding systemic treatment, there are three arms comparing no neoadjuvant treatment (arm A) vs. neoadjuvant TIP (arm B) vs. neoadjuvant chemoradiotherapy (radiation therapy + cisplatin).

In distant metastatic disease in patients with good performance status, TIP or TPF are frequently the first choice of treatment, although TIP was only evaluated in the neoadjuvant setting. The ORR with TPF was 38.5% and median OS of 7 months, but with grade 3 toxicities in 65% of patients (6). A less toxic two-drug regimen with cisplatin and 5-FU had an ORR of 32% and a median OS of 8 months (10). All the above results are from phase II trials, since there is no phase III trial in first or subsequent lines of treatment of metastatic penile SCC. No major advances have been made in recent years in this field. The most recent study with a different cytotoxic agent, vinflunine, showed a 27.3% ORR and 8.4 months OS (11). A phase II trial with gemcitabine and cisplatin, a widely used regimen in other advanced SCC, was completed, but the results were not published until this date (NCT00210041). In second-line treatment, a small phase II trial demonstrated an ORR of 20% and 5 months of median OS with paclitaxel in monotherapy (12).

## Genomic Landscape

**Figure 1:**
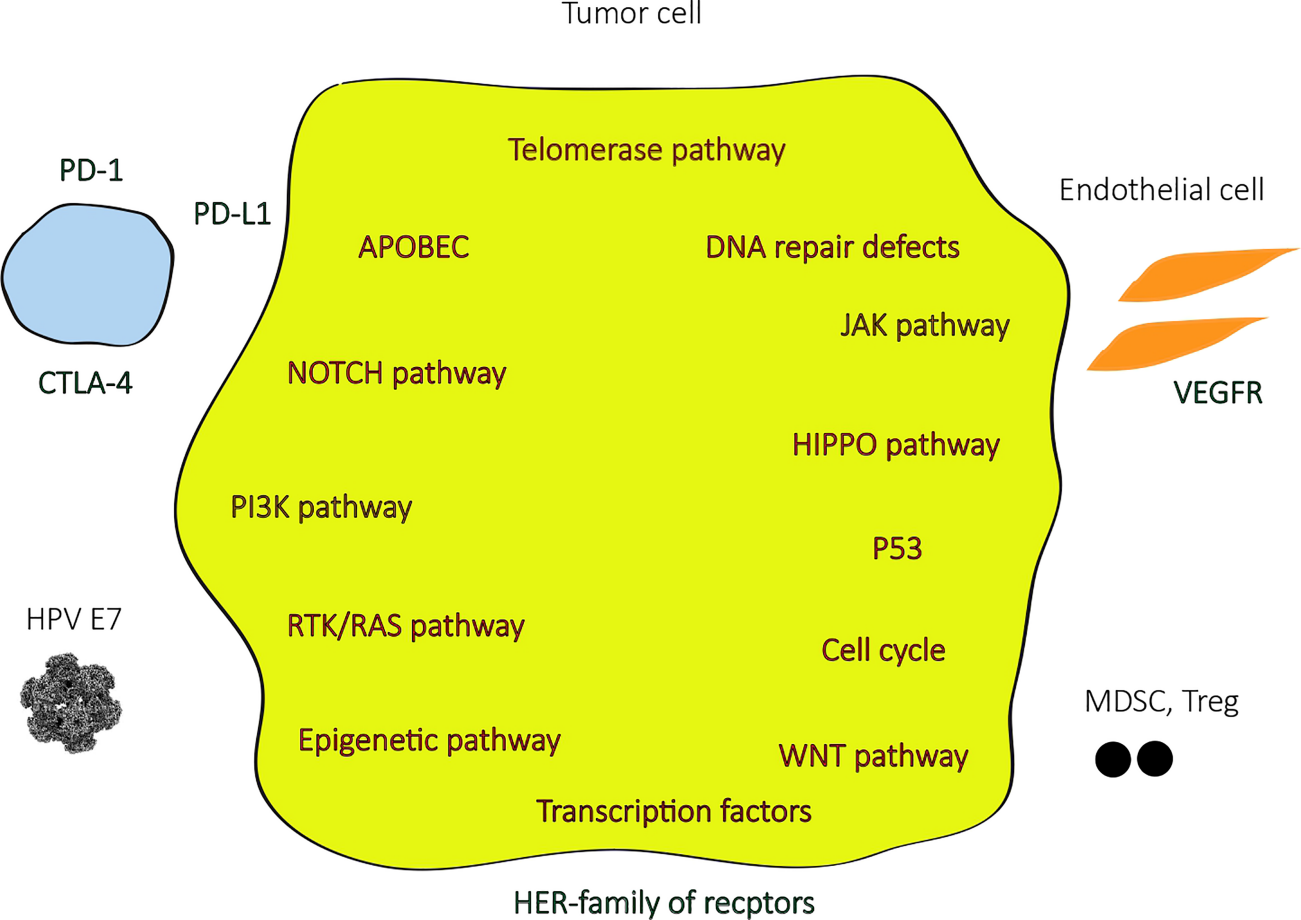
Genomic alterations, mutation signatures, and tumor microenvironment on penile SCC.

In the last few years, with the advances in next-generation sequencing (NGS) technologies, most of the genomic landscape of penile SCC became known (**Figure 1**), although the molecular signaling pathways and its role in carcinogenesis and tumor progression are yet to be better understood. Few studies have been reported from low-income countries, where the highest incidences of penile SCC are registered, especially in South America and Africa, and this can hamper a broader comprehension of the molecular pathogenesis of this disease. Some of the most relevant studies were analyzed in a very recent systematic review (13), where the most frequent somatic mutations found were TP53 (in up to 48%), CDKN2A, NOTCH1, PIK3CA, FAT1, CASP8, and FBXW7, and the most common copy number variations included gains in MYC (8q24) and amplification on EGFR (in up to 70% of cases). Amplifications or gains at HPV integration sites were identified in high frequencies (85 – 100%) in a single Brazilian study (14). The mutational burden was generally low and was found to be higher in HPV negative than in HPV positive associated penile SCC (15) and an even lower mutational burden was present in HPV positive malignancies with high viral load (16). HPV positive tumors were also associated with a lack of TP53 and CDKN2A mutations (15).

The most altered signaling pathways in penile SCC were NOTCH, RTK-RAS, and Hippo pathway (which frequently involves PIK3CA and EGFR alterations) in one recent study, which accounted for over 50% of tumors, and the frequently altered genes in these pathways were expressed in immunohistochemistry assay. RAS and Hippo pathways are potentially targetable with EGFR and mTOR inhibitors. Two mutation signatures were also identified in this study: the APOBEC-related signature, with a higher tumor mutational burden (TMB) with great potential to benefit from immunotherapy with checkpoint inhibitors, and the defective DNA repair system signature, which involves mutations in BRCA1, BRCA2, ARID1A, ATR, CHEK2, PARP1, FANCA, PALB2, and RAD51, a favorable scenario for treatment with immunotherapy and PARP inhibitors. The enrichment of NOTCH pathway alterations and the mutation signatures found in penile SCC in this trial were similar to head and neck SCC (17).

A study that performed comparative genomic profiling of refractory and metastatic penile and nonpenile cutaneous SCCs found a distinctive genomic pattern in penile SCC cases, including alterations in the mTOR pathway (NF1 and PTEN), in the DNA repair pathway (BRCA2 and ATM), and tyrosine kinase (EGFR, FGFR3, and ERBB2), all of them actionable therapeutic targets (18).

## Targeted and Anti-Angiogenic Agent Therapy

The EGFR family is important in penile SCC biology. One of the largest series, with 112 patients, showed that 44% had high expression of EGFR by immunohistochemistry, despite histologic subtype, histologic grade, or HPV status (19), and since KRAS mutations (which confers resistance to EGFR blockage in cancer treatment) are rare in these tumors (20), EGFR inhibitors have a potential role as systemic treatment. In a retrospective study, of 28 patients that received anti-EGFR monoclonal antibodies (cetuximab and panitumumab), alone or in combination with chemotherapy, 50% had a response to treatment and the median PFS was 3 months (21). One phase II trial with 11 advanced penile SCC pretreated patients that received panitumumab as a salvage therapy reached complete response in two patients and partial response in one, all of them with skin or lymph node metastasis, with a 1.9 months PFS and 9.5 months OS. Patients with visceral metastasis had no response. Grade 3 toxicity occurred in four patients (22). The NCCN lists monoclonal antibody EGFR inhibitors cetuximab or panitumumab as potential options based on modest datasets of retrospective and prospective studies demonstrating evidence of activity. The pan-HER (EGFR/HER1, HER2, and HER4) inhibitor dacomitinib produced a complete response in one and partial responses in eight of 28 patients (ORR 32.1%) in a single-arm phase II trial. The 12-month progression-free survival (PFS) was 26.2% and 12-month OS was 54.9% (23). The PENILANE trial (NCT02014831), a phase II with the association of cetuximab + TIP chemotherapy, active from 2013 to 2016, was withdrawn by the industry sponsor.

Vascular endothelial growth factor-A (VEGF-A) is the activating ligand of the VEGF receptor (VEGF-R), which plays a major role in cancer angiogenesis, and was overexpressed in 53.7% of penile SCC in a retrospective study (24). A small series of anti-angiogenic tyrosine kinase inhibitors sorafenib or sunitinib in six pretreated advanced penile SCC patients did not show exciting results. One partial response and four stable diseases were observed. Three patients showed pain response and had an improvement in quality of life (25). The phase II PAZOPEN-SOGUG trial (NCT02279576) that was evaluating the use of pazopanib with low doses of paclitaxel in advanced penile cancer was not completed due to its low recruitment.

## Immune Therapy

### Immune Checkpoint Inhibitors

Many HPV related cancers, with the similar histologic, epidemiologic, and therapeutic background to penile SCC, like head and neck, cervical, and anal carcinomas, have demonstrated good responses to immunotherapy with checkpoint inhibitors (26), due to its immunologic profile probably related to a higher mutational load and a high expression of PD-L1 (27). PD-L1 is expressed in 32.1% to 51.4% of penile cancer cells and 62.4% of tumor immune infiltrating cells and this biomarker is related to poor survival (28, 29).

Recently presented results of PERICLES phase II trial (NCT03686332), which included 32 patients with advanced penile cancer treated with atezolizumab, an anti-PD-L1 monoclonal antibody, alone or associated with local radiotherapy, showed a 30% oobjective response rate among 10 evaluable patients for response by RECIST 1.1 (including two complete responses), but the trial did not reach the expected PFS, its primary endpoint. Immunotherapy-related grade 3 or 4 adverse events occurred in 9.4% of patients (30). Avelumab, another anti-PD-L1 antibody already approved for the treatment of advanced urothelial cancer, is also under investigation in penile cancer as maintenance (NCT03774901) or second-line therapy (NCT03391479) after chemotherapy. Pembrolizumab, an anti-PD1 antibody already approved in a variety of advanced solid tumors, has shown durable responses (until 38 months) in case reports of penile cancer (31, 32), and the results of a prospective trial with this drug as monotherapy (NCT02721732) is expected (**Table 1**). This is a drug with a large experience in clinical practice and wide availability. Pembrolizumab is already US FDA approved for the agnostic treatment of high microsatellite instability (MSI-H) and high tumor mutational burden (TMB) ≥10 mutations/megabase in advanced solid tumors. However, the frequency of MSI-H penile SCC is very low (17) to translate the use of this drug commonly in practice following this criterion.

**Table 1:** Ongoing clinical trials in advanced penile cancer.

Status	Prior therapy required?	Tumors	Agent	Phase	Primary endpoint	n	ID
**Single agent immune checkpoint inhibitors**							
Active, not recruiting	yes	Rare tumors	Pembrolizumab	2	Non-progression rateIncidence of adverse events	202	NCT02721732
Recruiting	no	Advanced solid tumors	INCB099318	1	Number of treatment emergent adverse events	100	NCT04272034
Active, not recruiting	no	Penile Cancer	INCMGA0012 (Retifanlimab)	2	ORR	18	NCT04231981 (ORPHEUS)
Recruiting	no	Male genital tumors	LPD	2	pCR, ORR	127	NCT04718584
Recruiting	yes	Penile carcinoma	Avelumab (maintainance)	2	PFS	32	NCT03774901 (PULSE)
Recruiting	no	Penile carcinoma	Avelumab +/- radiotherapy	2	PFS	32	NCT03686332 (PERICLES)
Recruiting	yes	Penile carcinoma	Avelumab	2	ORR	24	NCT03391479
Recruiting	yes	Advanced solid tumors	XmAb20717	1	Safety and tolerability	154	NCT03517488 (DUET-2)
**Combinations of immune checkpoint inhibitors**							
Recruiting	no	Rare genitourinary tumors	Nivolumab + Ipilimumab	2	ORR	100	NCT03333616
Recruiting	yes	Rare tumors	Nivolumab + Ipilimumab	2	ORR	818	NCT02834013
Recruiting	yes	Advanced solid tumors	XmAb 22841 + Pembrolizumab	1	Safety and tolerability	242	NCT03849469 (DUET-4)
**Immune checkpoint inhibitors + chemotherapy**							
Recruiting	neoadjuvant	Penile Cancer	TIP + Nimotuzumab + Triprilimab	2	pCR	29	NCT04475016
Recruiting	no	Penile Carcinoma	Pembrolizumab + Cisplatin/Carboplatin + 5-FU	2	ORR	33	NCT04224740 (HERCULES)
**Immune checkpoint inhibitors + anti-angiogenicg agents**							
Recruiting	no	Rare genitourinary tumors	Nivolumab + Ipilimumab + Cabozantinib	2	ORR	224	NCT03866382
Active, not recruiting	yes	Genitourinary tumors	Nivolumab + Cabozantinib +/- Ipilimumab	1	Recommended phase II dose Incidence of adverse events	152	NCT02496208
Active, not recruiting	no	Rare solid tumors	Avelumab + Bevacizumab	2		137	NCT03074513
**Immune checkpoint inhibitors + epigenetic modifying agents**							
Recruiting	no	Advanced mucosal cancer	Pembrolizumab + Vorinostat	2	ORR	111	NCT04357873 (PEVOsq)
Recruiting	no	Virus-associated cancers	Avelumab + valproic acid	2	ORR	39	NCT03357757 (LATENT)
**HPV-directed therapies +/- combinations**							
Recruiting	yes	HPV-associated Squamous cell carcinomas	HB-201 and HB-202 (Arenavirus vectors)	1/2	Dose escalation Dose expansion	200	NCT04180215
Active, not recruiting	yes	HPV-associated cancers	DNA plasmids therapeutic vaccine MEDI0457 + Durvalumab	2	ORR	77	NCT03439085
Active, not recruiting	yes	Head and neck, cervical and penile squamous cell carcinomas	HPV anti-CD40 RNA vaccine	1/2	Safety and tolerability	44	NCT03418480 (HARE-40)
Completed	no	HPV-indeuced cancers	P16_37-63 peptide vaccination + cisplatin based chemotherapy	1	Immune response	11	NCT02526316 (VICORYX-2)
Active, not recruiting	no	HPV associated cancers	HPV specific T cells + Nivolumab	1	Safety and tolerability	32	NCT02379520 (HESTIA)
							NCT00019110
**Drug conjugate**							
Recruiting	yes	Advanced solid malignancies	PEN-866	1/2	Safety and tolerability ORR	340	NCT03221400

The combined therapy with two classes of checkpoint inhibitors, anti-PD-1/PD-L1 and anti-CTLA4 antibodies, can improve the response to immunotherapy, as the blockage of B7-CTLA-4 pathway leads to increased activation of CD8+ cells in the lymph nodes as well as increased infiltration of activated CD8+ T cells into the tumor, which enhances the antitumor immunity induced by anti-PD-1/PD-L1 drugs (33). The combination of nivolumab plus ipilimumab has demonstrated higher efficacy than monotherapy in advanced melanoma (34), lung cancer (35), renal cancer (36), hepatocellular carcinoma (37), and MSI-H colorectal cancer (38). A multi-cohort phase II trial investigated the combination of nivolumab and ipilimumab in 56 patients with advanced rare genitourinary cancers. Despite the 16% ORR in the entire cohort, there were, unfortunately, no objective responses among the five penile cancer patients, and only two stable diseases. Grade 3 or higher toxicity was observed in 39% of patients (39). Nivolumab plus Ipilimumab is being tested in penile cancer in one ongoing trial (NCT02834013) and this checkpoint inhibitors combination is in association with cabozantinib in two other ongoing trials (NCT03866382, NCT02496208) (**Table 1**).

### Immune Checkpoint Inhibitors Combined With Cytotoxic Chemotherapy

It is known that even with a minor response, cytotoxic chemotherapy is associated with tumor cell death and antigen shedding, which can be taken up by monocytes, macrophages, and dendritic cells and presented to T cells, initiating an antitumor immune response (40). Chemotherapy can also have an inhibitory effect on regulatory cells and myeloid suppressive cells (41). Immunotherapy with checkpoint inhibitors can enhance the response to chemotherapy by blocking the “silencing” signals of the immune response.

An association of pembrolizumab with cisplatin/carboplatin and 5-FU in inoperable and metastatic penile SCC is being evaluated on phase II LACOG 0218 trial (NCT04224740), which deserves special attention, as it is one of the few prospective studies underway in developing countries that have areas of higher incidence of this neoplasia (**Table 1**).

The single-center and single-arm phase II B2020-103-01 trial (NCT04475016) is evaluating the combination of TIP with nimotuzumab and triprilimab as a neoadjuvant treatment in locally advanced penile cancer (**Table 1**). Nimotuzumab is an intermediate affinity anti-EGFR antibody that inhibits cell proliferation and angiogenesis, activates natural killer cells, stimulates dendritic cell maturation, induces cytotoxic T cells, and restores MHC-I expression on tumor cells, hindering one of the EGFR immune-escape ways. In patients with locally advanced SCC of the head and neck, nimotuzumab in combination with low-dose cisplatin and radiotherapy was superior to cisplatin and radiotherapy in progression-free survival, disease-free survival, and locoregional tumor control (42). Triprilimab (JS001) is a recombinant humanized IgG4 anti-PD-1antibody that has demonstrated clinical activity in heavily pretreated nasopharyngeal cancer (43).

### Immune Checkpoint Inhibitors Combined With Anti-Angiogenic Agents

The association of checkpoint inhibitors and anti-angiogenic drugs is a well-known strategy that impacts on overall response rate and survival in another hypervascularized advanced urological tumors such as renal cell cancer (44, 45). Results of the phase I trial and expansion cohorts of Nivolumab, Ipilimumab, and Cabozantinib, a multitarget tyrosine kinase inhibitor (NCT02496208), presented at ASCO GU 2021, demonstrated an ORR of 44% in the penile SCC group of nine patients. The grade 3 or 4 treatment-related adverse events to the whole population of the study was 80% with the three-drug combination (46). Two phase II trials currently ongoing address this therapeutic approach, all of them basket trials including patients with penile SCC. One of them is also evaluating the combination of Nivolumab and Ipilimumab with Cabozantinib (NCT03866382), and the other one, the association of Atezolizumab and Bevacizumab, an anti-VEGF antibody (NCT03074513) (**Table 1**).

### Immune Checkpoint Inhibitors Combined With Epigenetic Modifying Agents

Although activity with immunotherapy is expected in penile SCC, similar to SCCs originating in other organs, there is a subset of tumors that presents with primary or secondary resistance to checkpoint inhibitors. One of these mechanisms of resistance is related to epigenetic processes that involve antitumor immunity pathways by affecting the antigenic presentation machinery and/or expression of the tumor antigen recognized by the immune system. The frequency of mutations in epigenetic modulator genes was found to be as high as 47% in SCCs (47). The Histone Deacetylases (HDAC) are a class of enzymes that play a crucial role of epigenetic modifications related to T cell differentiation and effector functions (48). The use of HDAC inhibitors can restore antigen presentation through an increase of TAP-1 and TAP-2, which allows the formation of the MHC I-peptide complex (49) and also increases PD-L1 expression (50). Vorinostat is an HDAC inhibitor that has shown a higher ORR when combined with pembrolizumab versus pembrolizumab alone (48% versus 25%, P = 0.026) in advanced PD-L1 > 1% NSCLC in the preliminary results of a phase II trial in 47 patients, with patients in the combination arm experiencing more fatigue, anorexia, and nausea, but with grade 3 or higher adverse events in only one out of 23 patients (51), while Etinostat, another HDAC inhibitor, associated with pembrolizumab, produced a 19% ORR in patients with metastatic melanoma pretreated with anti-PD-1/PD-L1 drugs (52). The combination of vorinostat and pembrolizumab is under investigation in a phase II basket trial of metastatic SCCs, including penile tumors (NCT04357873) (**Table 1**).

Other agents can lead to epigenetic modifications that enhance responses to therapy. Valproic acid has been demonstrated to enhance cisplatin-induced DNA damage through the downregulation of Excision Repair Cross-Complementing 1 (ERCC1), which is critical in DNA repair, and by increasing cisplatin influx and decreasing cisplatin export from human head and neck SCC cancer cells and decreases cetuximab-induced nuclear translocation of EGFR, a mechanism known to render chemotherapy resistance (53). Valproic acid also has an immunoregulatory activity through inhibition of histone deacetylases by decreasing the proportion of polymorphonuclear myeloid-derived suppressor cells (MDSCs) and attenuating the immunosuppressive function of these cells in patients with cancer. It was also found that valproic acid downregulates the expression of PD-L1 on MDSCs attenuating the suppressive effect of PD-1 on CD8+ T cells and promoting CD8+ T cells’ function (54). The ongoing phase II trial LATENT (NCT03357757) combines avelumab with valproic acid in the treatment of advanced viral-associated cancer (including penile SCC) (**Table 1**).

## Novel Therapeutic Targets

Heat shock proteins (HSPs) are molecular chaperones that function to maintain protein homeostasis through the proper folding and activation of client proteins in the cell and are characterized by their ability to become overexpressed under conditions of stress. HSP90 is one of the best understood of these proteins. Cancer cells are able to selectively modulate HSP90 activity through favorable complexes to satisfy the cells’ requirement to survive (55). A previous phase I study with a small-molecule inhibitor that targets HSP90 (PU-H71) showed objective responses in lymphomas and solid tumors, including 20.8% of tumor regression in a penile SCC patient (56).

PEN-866 is a miniature drug conjugate that targets and binds to activated tumor HSP90 protein and releases an SN-38 (an active metabolite of irinotecan) cytotoxic payload. This drug was well tolerated and demonstrated preliminary evidence of antitumor activity in a previous study (57). An ongoing phase I/IIa trial is investigating the role of PEN-866 in previously treated advanced solid malignancies, including penile SCC (NCT03221400) (**Table 1**).

M7824 is an innovative first-in-class bifunctional fusion protein composed of a human IgG1 monoclonal antibody against PD-L1 fused with two extracellular domains of TGF-bRII (a TGF-b “trap”) (58) that have demonstrated signs of efficacy in a phase I trial, with one complete response and partial responses in other cervical and anal cancer patients, that are HPV related tumors with histologic similarities to penile SCC. A phase I trial of M7824 in 16 patients with HPV associated malignancies showed a safety profile and a 37.5% ORR. The ORR in 11 HPV+ patients was 45.5% (59). There is a completed phase II trial with M7824 in the same subset of patients (NCT03427411), but the results were not published to date (**Table 1**).

A phase I trial with a small-molecule PD-L1 blocker, INCB099318, an oral drug, is ongoing and includes many advanced solid tumors, among which are penile SCCs (NCT04272034) (**Table 1**). This is an innovative administration of immunotherapy. Preliminary results of a phase I trial with a similar drug, INCB086550, reported in 2021, showed a similar toxicity profile to those seen with antibody immune checkpoint inhibitors, with the exception of a higher incidence of peripheral neuropathy (60).

XmAb22841 is a bispecific antibody that simultaneously targets immune checkpoint receptors CTLA-4 and LAG-3 that has a bispecific Fc domain to the two antigen-binding domains that confers long circulating half-life and stability and have been engineered to eliminate Fc gamma receptor (FcγR) binding, and can prevent the inhibitory action of some FcγR, avoiding resistance and improving the response to checkpoint inhibitors antibodies (61). The effect of this new drug is being studied in advanced solid tumors, including penile SCC, associated with pembrolizumab in the phase I trial DUET-4 (NCT03849469). Another bisepecific antibody, XmAb20717, which simultaneously targets PD-1 and CTLA-4, is also under investigation in a phase I trial (NCT03517488) for multiple types of advanced solid tumors, and preliminary results of 110 patients with a median of four previous systemic therapies (including immunotherapy checkpoint inhibitors in 64.5%) showed an ORR of 13% with very similar adverse events to anti-PD-1/PD-L1 antibodies (62) (**Table 1**).

## HPV-Directed Therapies

Human papillomavirus (HPV) is strongly implicated in penile SCC carcinogenesis, although exact pathways are not completely understood, and is an important area of interest regarding tumor prevention and treatment of this neoplasia, as well as in other HPV-related neoplasia such as cervical cancer, where it is better established. Approximately 20% to 50% of penile cancer is driven by HPV infection (63). The largest analyzed sample relies on a systematic review of 1266 invasive penile SCC patients in North America and reported that up to 48.7% of penile SCC harbors HPV DNA (64). Differently, we can find a higher proportion of HPV positive tumors in populations with a higher incidence as in northeast Brazil, where a study with 55 patients found that 89.1% of samples were positive for HPV DNA (65). The majority of the HPV infection in penile SCC is represented by the high-risk subtypes 16 and 18 (30.8% and 6.6%, respectively) (66). HPV positive tumors have a better prognosis than HPV negative tumors and PD-L1 expression is higher in HPV negative than in HPV positive penile SCC (49.4 vs. 32.7%, respectively, p = 0.03) (67). Preclinical studies in head and neck SCC suggest that the use of the HPV vaccine can upregulate PD-1 acting as a synergistic therapy with PD-1 checkpoint inhibitors to enhance antitumor efficacy (68).

Patients with cervical intraepithelial neoplasia 2/3 were treated with a therapeutic synthetic DNA vaccine targeting human papillomavirus 16 and 18 E6 and E7 proteins in a phase IIb trial and had a significantly higher histologic regression when compared to placebo (48.2% vs. 30%, respectively, p = 0.034) demonstrating that it is possible to block the progression to malignant tumors using an anti-viral immunotherapy (69). However, in HPV16-positive advanced or recurrent gynecological carcinoma, an HPV16 synthetic long peptide vaccine produced an immune response, but no tumor regression (70), suggesting that the action of vaccine-activated T cells on invasive tumors is blocked by a tumor-induced immunosuppressive microenvironment.

The association of an HPV vaccine and a checkpoint inhibitor was evaluated in a single-arm phase II trial that enrolled 24 patients with incurable HPV-16-positive cancer, most of them with oropharyngeal cancer, treated with ISA101, a synthetic long-peptide HPV-16 vaccine, and nivolumab. The ORR was 33%, median OS of 17.5 months and five patients had durable responses. Grade 3 toxicity was observed in two patients (71).

Ongoing trials of HPV vaccines, which include penile cancer patients, are a phase I/II trial of an HPV Anti-CD40 RNA Vaccine (HARE-40) (NCT03418480); a phase I trial of vaccine with human papillomavirus 16 E7 peptide and synthetic human papillomavirus 16 E6 peptide (NCT00019110); a phase I trial with a P16_37-63 peptide vaccine combined or not with ISA 51 VG (an emulsion with immunomoadjuvant activity that enhancing the cytotoxic T-lymphocyte response against antigens in vaccines) and associated with cisplatin-based chemotherapy (NCT02526316); phase II trial combining Durvalumab (an anti-PD-L1 antibody) with the DNA Plasmid-encoding Interleukin-12/HPV DNA Plasmids Therapeutic Vaccine MEDI0457 (NCT03439085); a phase I/II trial of treatment of HPV16+ cancers with arenavirus vectors HB-201 and HB-202, that expresses the same non-oncogenic HPV16 E7E6 fusion protein and induces tumor-specific T-cell responses (NCT04180215) (**Table 1**). In this last trial, in a preliminary analysis, two of 11 evaluable patients treated with HB-201 had a partial response and six had stable disease, with a duration of response of 4.8 months. All six evaluable patients that received HB-201/HB-202 had stable disease and serious adverse events related to treatment occurred in 24% of patients (72).

Adoptive T-cell therapy (ACT) is also a promising cancer treatment modality that is showing encouraging results in clinical trials. Infusion of tumor-infiltrating T cells preceded by a lymphocyte-depleting conditioning regimen and followed by systemic high-dose aldesleukin was performed in 29 patients with metastatic HPV related cancers (18 cervical and 11 non-cervical). Objective tumor responses occurred in 28% of patients in the cervical cancer cohort and 18% of patients in the noncervical cancer cohort. Two of the responses in cervical cancer were complete and are ongoing 67 and 53 months after treatment. Responses in the noncervical cancer cohort were in anal cancer and oropharyngeal cancer. There were no acute infusion-related toxicities and no autoimmune adverse events (73).

Successful expansion of tumor-reactive tumor-infiltrating lymphocytes (TIL) from lymph nodal metastasis of penile cancer patients, with 46.8% of CD8+ T cells and 45.4% from expanded TIL secreting IFN-γ in response to autologous tumor, supports the development of ACT strategies using TIL for the treatment of advanced and recurrent penile cancer (74).

Patients with penile cancer are currently included in the eligibility criteria of the HESTIA trial, a phase I trial using HPV-specific T cells collected from the blood of patients with HPV cancers associated with nivolumab (NCT02379520) (**Table 1**).

## Conclusions

Despite its rarity, advanced penile cancer is an important health issue, considering the poorer prognosis compared to early disease which is curable with surgery alone, and the absence of a highly efficient standard systemic treatment. Cytotoxic chemotherapy remains the mainstay of treatment, even though it is based on small phase II trials, due to the lack of trials with a higher level of evidence. Toxicity with chemotherapy combination regimens is high to the point that about half of patients experience a grade 3 adverse event.

A better knowledge of the genomic landscape and immune microenvironment of penile SCC demonstrated similarities with head and neck SCC and allowed the development of clinical trials with different modalities of systemic treatment. Alterations in NOTCH, RTK-RAS, Hippo, mTOR, and DNA repair pathways offer actionable targets with potential for new treatments. High T cell infiltration and expression and PD-L1 in a large part of these tumors led to trials with a variety of immune checkpoint inhibitors, alone or in combination with other immunotherapies, cytotoxic drugs, or targeted therapies, with favorable preliminary results for some of them. Positivity for HPV infection is also propitious to HPV-directed therapies, like vaccines and adoptive T-cell therapy, since they have been demonstrated to have good preliminary results with other HPV-associated cancers. However, most of these studies are basket trials and include a wide range of rare tumors with similar molecular alterations, for the extreme difficulty to recruit patients precludes the execution of large prospective trials in penile cancer exclusively.

The better way to increase accrual and consequently improve clinical outcomes resides in global collaborative studies, including centers located in proportionally higher incidence areas. Additionally, a paradigm of decentralized accrual of patients and global retrospective studies may be necessary to make advances, which will require an extremely collaborative effort with multiple stakeholders involved. Scientific collaboration is also the key to a deeper knowledge of the different genomic and epigenomic alterations in HPV positive and negative tumors, in addition to the development and sharing of penile SCC cell lines and animal models in order to boost a more profound comprehension of the tumor biology and more accurate planning of future trials.

## Author Contributions

All authors listed have made a substantial, direct, and intellectual contribution to the work, and approved it for publication.

## Conflict of Interest

The authors declare that the research was conducted in the absence of any commercial or financial relationships that could be construed as a potential conflict of interest.

## Publisher’s Note

All claims expressed in this article are solely those of the authors and do not necessarily represent those of their affiliated organizations, or those of the publisher, the editors and the reviewers. Any product that may be evaluated in this article, or claim that may be made by its manufacturer, is not guaranteed or endorsed by the publisher.
